# The Effect of Hyperoxia on Central and Peripheral Factors of Arm Flexor Muscles Fatigue Following Maximal Ergometer Rowing in Men

**DOI:** 10.3389/fphys.2022.829097

**Published:** 2022-02-03

**Authors:** Stefanos Volianitis, Peter Rasmussen, Nicolas C. Petersen, Niels H. Secher

**Affiliations:** ^1^Department of Physical Education, College of Education, Qatar University, Doha, Qatar; ^2^Department of Anesthesiology, Rigshospitalet, Institute for Clinical Medicine, University of Copenhagen, Copenhagen, Denmark; ^3^Department of Neuroscience, Faculty of Health and Medical Sciences, University of Copenhagen, Copenhagen, Denmark

**Keywords:** rowing, cerebral oxygenation, hyperoxia, maximal voluntary contraction, transcranial magnetic stimulation

## Abstract

**Purpose:**

This study evaluates the effect of hyperoxia on cerebral oxygenation and neuromuscular fatigue mechanisms of the elbow flexor muscles following ergometer rowing.

**Methods:**

In 11 competitive male rowers (age, 30 ± 4 years), we measured near-infrared spectroscopy determined frontal lobe oxygenation (ScO_2_) and transcranial Doppler ultrasound determined middle cerebral artery mean flow velocity (MCA *V*_mean_) combined with maximal voluntary force (MVC), peak resting twitch force (*P*_tw_) and cortical voluntary activation (VA_TMS_) of the elbow flexor muscles using electrical motor point and magnetic motor cortex stimulation, respectively, before, during, and immediately after 2,000 m all-out effort on rowing ergometer with normoxia and hyperoxia (30% O_2_).

**Results:**

Arterial hemoglobin O_2_ saturation was reduced to 92.5 ± 0.2% during exercise with normoxia but maintained at 98.9 ± 0.2% with hyperoxia. The MCA *V*_mean_ increased by 38% (*p* < 0.05) with hyperoxia, while only marginally increased with normoxia. Similarly, ScO_2_ was not affected with hyperoxia but decreased by 7.0 ± 4.8% from rest (*p* = 0.04) with normoxia. The MVC and *P*_tw_ were reduced (7 ± 3% and 31 ± 9%, respectively, *p* = 0.014), while VA_TMS_ was not affected by the rowing effort in normoxia. With hyperoxia, the deficit in MVC and *P*_tw_ was attenuated, while VA_TMS_ was unchanged.

**Conclusion:**

These data indicate that even though hyperoxia restores frontal lobe oxygenation the resultant attenuation of arm muscle fatigue following maximal rowing is peripherally rather than centrally mediated.

## Introduction

Maximal rowing provokes disturbance to systemic and intramuscular homeostasis (Volianitis and Secher, 2009; Volianitis et al., 2018) that exacerbates pulmonary diffusion limitations and reduces arterial hemoglobin oxygen saturation (SaO_2_) to below 88% (Nielsen et al., 1998). Such pronounced arterial hypoxemia, combined with the hyperventilation-induced reduction in PaCO_2_ by 8–10 mm Hg (Volianitis et al., 2008) and dependent cerebral blood blow (CBF), compromises oxygen cerebral delivery and reduces cerebral mitochondrial oxygen tension by more than 10 mm Hg (Nybo and Rasmussen, 2007) that can impair motor performance (Rasmussen et al., 2007). Conversely, when cerebral oxygenation is enhanced with oxygen supplementation rowing performance is improved (Nielsen et al., 1999), suggesting that the performance improvement may be attributed to attenuation of “central fatigue” (Gandevia, 2001), that is, enhanced volitional motor output to locomotor muscles (Nybo and Rasmussen, 2007). Alternatively, the ergogenic effect may stem from enhanced force-generating capacity of the exercising muscles due to processes at, or distal to, the neuromuscular junction (i.e., attenuation of “peripheral fatigue”; Allen et al., 2008), secondary to enhanced arterial oxygen content (CaO_2_) and hence oxygen delivery to muscles (Amann et al., 2006a). Oxygen supplementation attenuates the rate of development of peripheral fatigue provoked both by high intensity whole body exercise (Amann et al., 2006b; Romer et al., 2006; Dominelli et al., 2017) and isolated muscle exercise (Katayama et al., 2007), indicating that the effect is independent of possible attenuation of fatiguing metabolites, secondary to a hyperoxia-induced increase in maximal exercise capacity and, thus, changes in relative work intensity.

The contribution of both central and peripheral fatigue mechanisms is considered when attempting to explain performance fatigability, albeit implications for performance should be approached with caution, as the translation of fatigue mechanisms to human whole body performance is not straightforward (Enoka and Duchateau, 2016). The contribution of central and peripheral mechanisms to neuromuscular fatigue depends on the tested muscle group (Enoka et al., 2011) as shown for the upper and lower limbs (Vernillo et al., 2018), for example, 2 min MVCs result in central fatigue of the lower limbs, but not of the upper limbs indicating that fatigue mechanisms may be regulated differently and limb specific. The functional significance of such differential contribution of central activation to the force production of different muscle groups can be appreciated with the leg “strength paradox,” that is, the strength deficit of a bilateral leg effort, typically 15–20%, compared to the strength expected from the sum of the separate unilateral leg efforts, as observed in rowers (Secher, 1975).

Rowing is unique in using both arms and legs in a synchronous manner (i.e., bilateral leg extension and arm flexion), as opposed to most sports and daily life where the limbs are used alternatively as in, for example, walking and running. The training adaptation of such synchronous movement is reflected in attenuation of the strength deficit of bilateral leg effort in rowers. Indeed, in the very best trained rowers the bilateral leg strength may even surpass the sum of the strength of each leg (Secher, 1975). The comparison between unilateral vs. bilateral leg efforts allows for investigation of differences in muscle mass-related fatigability, as suggested by Rossman et al. (2012) and Koral et al. (2020). In contrast to leg efforts, there is no “strength paradox” when using the arms, that is, the strength measured during simultaneous use of both arms corresponds to the sum of the strength of each arm determined separately (Secher et al., 1988). Taken together, the seemingly differential central motor activation to upper and lower limbs suggests that the legs may be more prone to develop force deficit than the arms in circumstances where central motor activation is challenged, as in maximal rowing. In support, leg strength deficit following maximal rowing has been attributed to supraspinal origin (Husmann et al., 2017). However, data on arm neuromuscular activation following rowing are lacking.

This study evaluated the contribution of central and peripheral factors to elbow flexors fatigue following maximal rowing. Considering the different contribution of centrally and peripherally located fatigue mechanisms of upper and lower limbs (Vernillo et al., 2018), we hypothesized that central factors would be less prevalent compared to peripheral factors in the arms following maximal ergometer rowing. In addition, considering the marked arterial desaturation developed during rowing, a second aim of this study was to evaluate the effect of oxygen supplementation on central vs. peripheral factors of elbow flexor muscle fatigue. A second hypothesis was that oxygen supplementation would attenuate central fatigue by enhancing cerebral oxygenation. Maximal voluntary contraction (MVC), transcranial magnetic stimulation (TMS) of the motor cortex, and electrical stimulation of the motor point (MP) were applied for evaluation of central and peripheral factors of elbow flexor muscles fatigue. Also, CBF, cerebral (ScO_2_) and muscle oxygenation (SmO_2_) evaluations were applied as factors associated with rowing fatigability.

## Materials and Methods

### Participants

Eleven healthy males (mean ± SD age, 30 ± 4 yrs.; height, 1.80 ± 0.03 m; weight, 75.0 ± 3.1 kg), following written informed consent, volunteered to the study as approved by the Ethics Committee of Copenhagen (KF 01287471) and conformed to the Declaration of Helsinki. Sample size calculation was based on changes in cortical voluntary activation following maximal dynamic exercise (∆ VA_TMS_, −13%; Rasmussen et al., 2010) and the typical error for the measurement of elbow flexion MVC (ICC, 0.992; Allen et al., 1995) with power of 0.80 and an *α* of 0.05. All subjects were recruited from a local rowing club and were well familiarized with maximal ergometer rowing as they had been competing for several years at national/international level (one was a current World champion and two others were members of the national team).

### Experimental Design

Each subject completed one familiarization and two experimental trials. During the familiarization trial, subjects practiced maximal voluntary isometric elbow flexion with and without TMS and electrical stimulation. “All-out” 2,000 m rowing on a wind-braked ergometer (Concept II, Morrisville, VT, United States) was performed with normoxia [inspiratory O_2_ fraction (FIO_2_), 0.21] and hyperoxia (FIO_2_, 0.30), in a pseudo-randomized counter-balanced order, in two experimental trials separated by 1–2 weeks at the same time of day under consistent laboratory conditions (temperature 22 ± 1°C, humidity 50 ± 10%, barometric pressure 757 ± 4 mmHg). While the conditions were not blinded, the participants were naive to the purpose of the study and unaware of the experimental hypotheses. The subjects refrained from strenuous exercise, alcohol, and caffeine for 24 h prior the investigations and reported to the laboratory after an 8 h overnight fast. On each trial, the subjects rowed for about 20 min on the ergometer with an individually chosen pre-race warm-up that were accustomed. Then, submaximal and maximal contractions (60–100% MVC) were performed to allow estimation of resting twitch for the calculation of VA_TMS_, and the stimulation intensities for MP and TMS were determined (for details see the Neuromuscular Evaluation section). Then, the subjects performed two 4-s MVCs, separated by 2 min of rest, while still seated on the rowing ergometer. If the difference between these MVCs was >5%, a third MVC was performed. The largest MVC was taken to represent control muscle strength. For all MVCs subjects were encouraged to apply maximal effort and to try to maintain the same intensity throughout the 4-s period. Hyperoxic air was humidified in a Douglas bag and delivered to the subjects through a two-way low-resistance T valve (model 2,700, Hans Rudolph, Kansas City, MO, United States) for 5 min prior to and during rowing. Breath-by-breath O_2_ consumption (VO_2_) and ventilation (VE) were measured with an online gas analyzer (CPX/D, Medical Graphics, St. Paul, MN, United States) and data averaged over 30 s. During rowing, the subjects were verbally encouraged to perform maximally.

Blood samples were drawn anaerobically from a catheter (20 gauge; 1.1 mm) inserted in the radial artery of the non-dominant arm (left, for all subjects) three times at rest, once after 500, 1,000, 1,500 m of rowing and immediately after exercise in heparinized syringes and analyzed immediately for blood gas variables (ABL 725; Radiometer, Copenhagen, Denmark).

### Cerebral Perfusion

Transcranial Doppler ultrasound (2 MHz probe; Multidop X; DWL, Sipplingen, Germany) determined middle cerebral artery mean flow velocity (MCA *V*_mean_) as an index of CBF, since changes in MCA *V*_mean_ reflect those of CBF during dynamic exercise (Secher et al., 2008). The MCA *V*_mean_ was the mean velocity of the time-averaged maximal velocity over the cardiac cycle derived from the envelope of the maximum frequencies of the Doppler spectra. The MCA was located by insonation through the temporal ultrasound window and the position with the highest signal to noise ratio (depth 48–60 mm) was marked. The probe was fixed to a headband with adhesive sonography gel and data sampled at 100 Hz (Chart v5.2 and PowerLab; ADInstruments, Bella Vista, NSW, Australia).

### Cerebral and Muscle Oxygenation

ScO_2_ and SmO_2_ were evaluated using near-infrared spectroscopy (NIRS; INVOS 5100C, Somanetics, Troy, MI, United States). For ScO_2,_ the optode (3 and 4 cm emitter detector separation, wavelength 730 and 808 nm) was placed over either the right or left prefrontal cortical area, in randomized order, between Fp1 and F3, or Fp2 and F4, according to the landmarks of the 10–20 system (Perrey, 2008) to avoid influence from the frontal and sagittal sinus and ipsilateral to the Doppler probe. This area of the brain is not directly involved in the neural control of movement, but deoxygenation of the prefrontal cortex has been associated with termination of exercise during both controlled and self-paced exercise (Nielsen et al., 1999; Amann et al., 2007; Seifert et al., 2009; Subudhi et al., 2009).

For SmO_2_ the optode was positioned on the vastus lateralis muscle of the left leg at the midpoint between the anterior superior iliac spine and the superior part of the fibula. Hair on the leg was removed for maximal optode contact and the same position of the probe was used in both trials. We considered that reductions in SmO_2_ and ScO_2_ together with cerebral perfusion would be indicative of peripheral and central fatigue, respectively.

### Neuromuscular Evaluation

**Figure 1:**
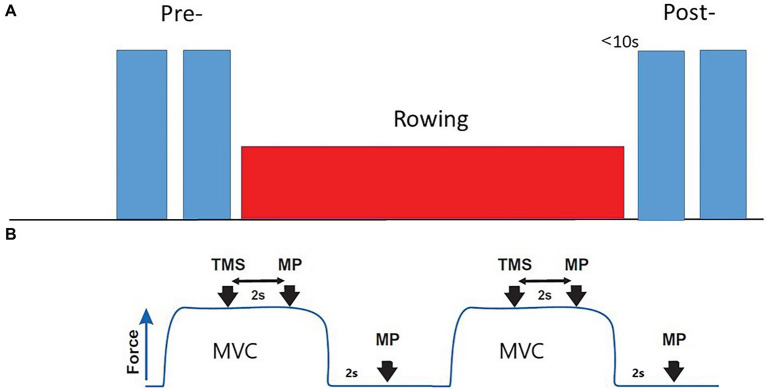
Neuromuscular evaluation protocol. **(A)** Pre- baseline evaluation; Post-, After rowing (less than 10 s) evaluation. **(B)** Each evaluation included two 4 s MVCs. One TMS and one MP stimulation, interspersed by 2 s, followed by a resting MP stimulation 2 s after the end of contraction, were applied with each MVC.

Neuromuscular evaluation was performed after the warm-up and immediately after the rowing trials (Figure 1). The transition time from the end of the rowing to the start of the neuromuscular evaluation was less than 10 s. It was important to minimize this transition time because neuromuscular fatigue is strongly influenced by recovery, as all parameters recover almost linearly within 1 min (Vernillo et al., 2018). With this consideration, the subjects’ right arm was swiftly attached to a custom designed arm-bar equipped with a calibrated strain gauge dynamometer (14-bit A/D conversion) while they were still seated on the ergometer. The shoulder and elbow of the subjects were flexed at 90° with the forearm vertical and fully supinated. The dynamometer’s position was adjusted in direct line with the applied force and secured at the wrist. The neuromuscular evaluation included two 4-s MVCs for determination of (averaged) elbow flexors strength, with one TMS and one motor point (MP) stimulation superimposed on each MVC, followed by another resting MP stimulation 2 s after each MVC to obtain peak potentiated twitch force (*P*_tw_). Electromyographic (EMG) activity was recorded with pairs of self-adhesive surface (10-mm recording diameter) electrodes (Cleartrace, 1700-030, Conmed, Utica, NY, United States) placed over the muscle belly and tendons of biceps brachii, brachioradialis, and long head of triceps brachii muscles, in bipolar configuration with a 30-mm interelectrode distance and the reference on the medial epicondyle of the humerus. Low impedance (<5 kΩ, controlled by a digital meter) between electrodes was achieved by shaving and gently abrading the skin and then cleaning it with isopropyl alcohol. The positions of EMG electrodes were marked with indelible ink to ensure consistent placement. The EMG signal was amplified (×1,000), filtered (25–1,000 Hz), digitized, and sampled (at 5 kHz) to a computer using CED Micro1401 and Spike2 software (Cambridge Electronic Design, Cambridge, United Kingdom). The EMG activity was quantified as the root-mean-square value and expressed relative to the activity obtained during the control MVC.

#### Brachial Plexus Stimulation

Before the voluntary contractions, the size of the resting maximal compound muscle action potential (Mmax) was determined with surface EMG and used as a reference for the size of the EMG responses evoked by TMS. While the subjects were at rest single electrical stimuli of 100-μs duration were delivered to the brachial plexus *via* a cathode in the supraclavicular fossa (Erb’s point) and an anode on the acromion. The electrical current was gradually increased until the M-wave (i.e., Mmax) of the biceps brachii no longer increased. A supramaximal stimulation current (i.e., 20% higher than that required to elicit Mmax) was used for the remainder of the experiment. The supramaximal stimulus intensitys was 136 ± 52 mA.

#### Transcranial Magnetic Stimulation

Single magnetic pulses to the motor cortex were delivered with a circular coil (13.5 cm outside diameter; Magstim 200, The Magstim Co. Ltd., Whitland, Wales, United Kingdom) with its center placed at the vertex, and thus evoked motor potentials (MEPs) in the biceps brachii. The vertex was determined by marking the intersection of the measured halfway points from nasion to inion and from tragus to tragus. The optimal coil position was the site where the largest MEP was elicited, and it was marked on the scalp for consistent positioning throughout the protocol. The direction of the current flow in the coil was clockwise on the left motor cortex (postero-anterior intracranial current flow) to activate preferentially the muscles on the contralateral side. The TMS intensity was determined from a stimulus–response curve constituted of four brief consecutive contractions at 50, 60, 70, and 80% maximal stimulator output, in randomized order. The selected stimulus intensity was the lowest intensity eliciting maximal MEP amplitudes (>50% Mmax) in m. biceps brachii (with little or no response, usually <10–15% of Mmax, in the antagonist m. triceps brachii) during brief voluntary contractions at 20% MVC. The TMS was always delivered once the voluntary contraction reached the intended force level and the force had stabilized.

#### Electrical Stimulation

For electrical stimulation of the biceps muscle, surface electrodes (Cleartrace, Conmed) were placed on each of the MPs approximately halfway from the coracoid process to the lateral epicondyle of the humerus (Park et al., 2007). A computer triggered a double 1 ms electrical stimulus at constant current (inter-stimuli interval: 10 ms, DS7A Digitimer, Hertfordshire, United Kingdom). The maximal resting twitch was determined by stepwise increases in the stimulus intensity until elbow flexor twitch force failed to increase, despite an increase in stimulus intensity. Stimulation intensity was set 10–20% above the level required to produce a resting twitch of maximal amplitude and it was 122 ± 32 mA.

#### Cortical Voluntary Activation (VA_TMS_)

**Figure 2:**
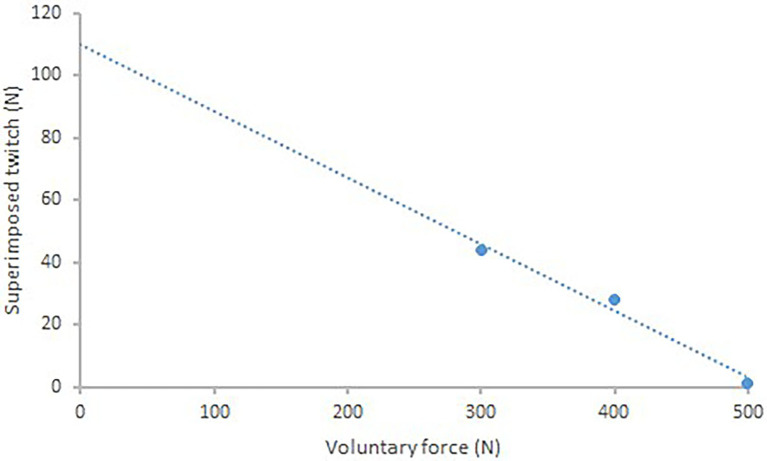
Single subject data showing linear regression between the amplitude of the TMS superimposed twitch and voluntary force (*r* = 0.99). The *y*-intercept (114.5 N) is taken as the estimated amplitude of the resting twitch.

Cortical voluntary activation was quantified by the force responses. Using magnetic cortical stimulation any increment in elbow flexion force evoked during an MVC (superimposed twitch) was expressed as a fraction of the amplitude of the maximal response evoked by the same stimulus in the relaxed muscle. The resting twitch was estimated by linear interpolation of the TMS induced muscle twitches obtained at 60, 80, and 100% of MVC and was identified as the y-intercept of the regression corresponding to the value at which voluntary force would be zero (Figure 2; Todd et al., 2003):


$$\mathrm{Voluntary}\;\mathrm{activation}\;\left(\%\right)=\left(\frac{1-\mathrm{superimposed twitch}}{\mathrm{estimated resting twitch}}\right)\times 100$$

Data points were excluded (*n* = 5.4%) when the regression of the estimated twitch was *r*^2^ < 0.85.

The reliability of the TMS protocol for the determination of voluntary activation and estimated resting twitch (ICC < 0.85) is comparable to values derived from motor nerve stimulation (Sidhu et al., 2009).

### Statistics

Assumptions of sphericity (Mauchly test) and normality (Shapiro–Wilk test) were tested for all dependent variables. If the assumption of sphericity was violated, the corrected value for non-sphericity with Greenhouse–Geisser epsilon was reported. Values are presented as mean ± SD.

A two-way analysis of variance (ANOVA) with repeated measures (time × trial) was used to reveal significant interactions between conditions and the Tukey *post hoc* test for paired data was used to locate differences. F-ratios were considered statistically significant at *p* < 0.05 level and analysis was performed using SPSS Statistics 24 (IBM, Armonk, NY, United States). Descriptive statistics in the text include mean percentage change for each dependent variable, while the reported *p* values are based on statistical comparison using absolute values.

## Results

In normoxia, the subjects completed the ergometer row in 6 min 56 ± 5 s and hyperoxia had no significant effect compared to normoxia (6 min 54 ± 4 s, 0.5% improvement). Yet, VO_2_ was 10.6% higher during the hyperoxic compared with the normoxic trial (*p* < 0.01, Table 1). Also, the reduction of SaO_2_ observed during the normoxic trial was prevented during the hyperoxic trial (*p* < 0.01, Table 1).

**Table 1:** Cerebral, cardiovascular, and respiratory variables at rest and over the last 30 s of rowing with normal and hyperoxic air.

	21% O_2_		30% O_2_	
	Rest	Rowing	Rest	Rowing
HR, beats min^−1^	71 ± 5	181 ± 2^*^	68 ± 2	178 ± 2^*^
MAP, mmHg	96 ± 2	104 ± 4^*^	94 ± 2	109 ± 3^*^
VE, L min^−1^	13 ± 1	168 ± 3^*^	13 ± 1	172 ± 5^*^
VO_2_, L min^−1^	0.4 ± 0.0	4.7 ± 0.2^*^	0.4 ± 0.0	5.2 ± 0.2^*^
PaO_2_, mmHg	103 ± 2	94 ± 2^*^	164 ± 3	161 ± 5^†^
PaCO_2_, mmHg	39 ± 1	29 ± 1^*^	39 ± 1	31 ± 1^*^
SaO_2_, %	98.3 ± 0.2	92.5 ± 0.2^*^	99.4 ± 0.1	98.9 ± 0.2^†^
MCA *V*_mean_, cm s^−1^	52.3 ± 8.2	54.7 ± 7.6	44.7 ± 4.0	61.7 ± 7.7^*^^†^
ScO_2_, %	67.3 ± 8.2	60.3 ± 5.4^*^	66.6 ± 3.6	67.7 ± 5.4^†^
SmO_2_, %	63.5 ± 7.3	41.2 ± 2.0^*^	64.9 ± 3.6	48.0 ± 3.4^*^^†^

HR, heart rate; MAP, mean arterial pressure; VE, ventilation; VO_2_, oxygen uptake; PaO_2_, arterial oxygen tension; PaCO_2_, arterial carbon dioxide tension; MCA *V*_mean_, middle cerebral artery mean blood flow velocity; SaO_2_, arterial oxygen saturation; ScO_2_ frontal lobe oxygen saturation; SmO_2_, oxygenation of the vastus lateralis muscle; Values are mean ± SD, *N* = 11.

* Difference compared with rest.

† Difference compared with normoxia (*p* < 0.05).

### Cerebrovascular Variables

**Figure 3:**
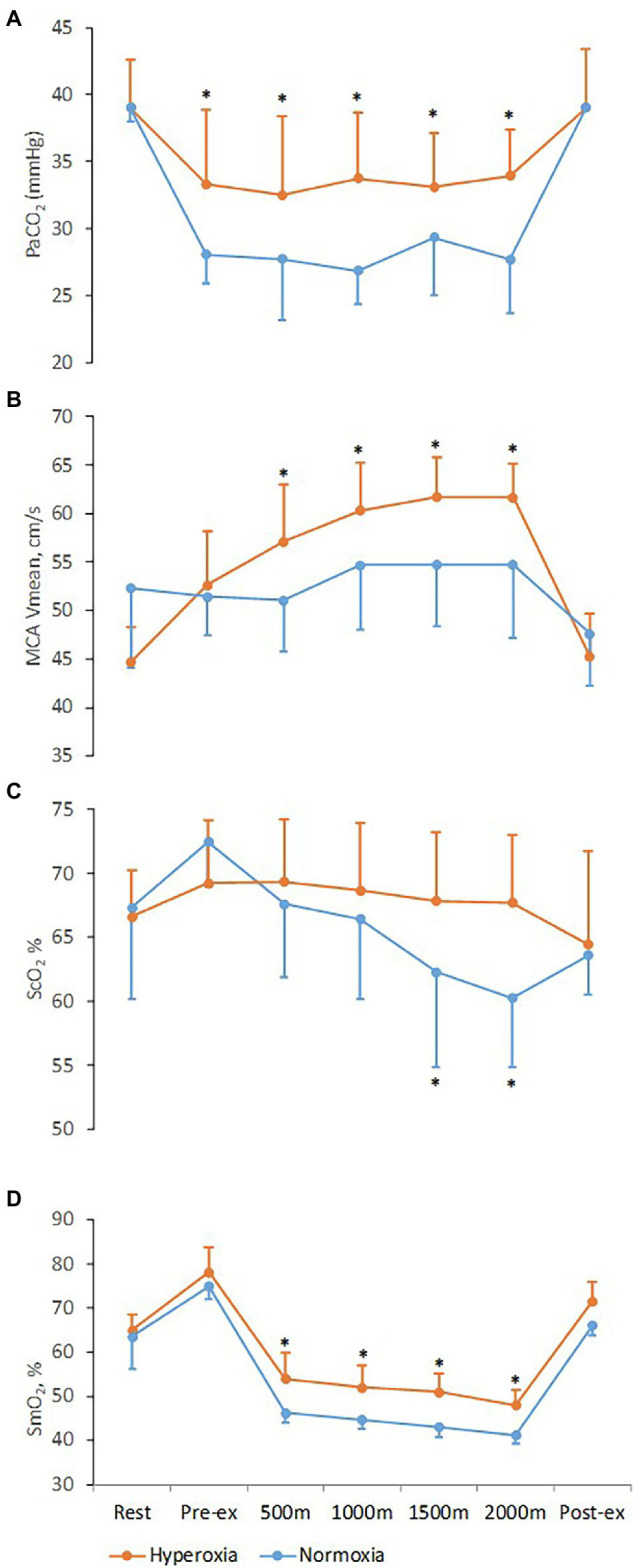
Respiratory, cerebrovascular, and muscle variables at rest and during 2,000 m all-out ergometer rowing with normoxia and hyperoxia. Pre-ex and post-ex values are obtained after the warm-up and 15 min into the recovery, respectively. **(A)** PaCO_2_, arterial carbon dioxide tension; **(B)** MCA *V*_mean_, middle cerebral artery mean blood flow velocity; **(C)** ScO_2_, frontal lobe oxygen saturation; **(D)** SmO_2_, oxygenation of the vastus lateralis muscle. Values are means ± SD; *Difference between normoxic and hyperoxic trials (*N* = 11, *p* < 0.05).

There was a significant interaction [*F*_(1, 12)_ = 5.9, *p* < 0.01] between trials and time for MCA *V*_mean_ and ScO_2_. During the normoxic trial MCA *V*_mean_, after a 4.6% increase by 1,000 m, plateaued until the end of the trial and returned to baseline after exercise (Figure 3; Table 1). During the hyperoxic trial, MCA *V*_mean_ increased from rest until 1,500 m of rowing (38% higher, *p* < 0.01), then plateaued until the end and also returned to resting level after exercise. The ScO_2_ decreased by 7.0 ± 4.8% (*p* < 0.01) during the normoxic trial, while it was maintained at resting level during the hyperoxic trial (Figure 3; Table 1).

### Muscle Oxygenation

There was interaction [*F*_(1, 12)_ = 5.4, *p* < 0.01] between trials and time for SmO_2._ During the normoxic trial, SmO_2_ was reduced by 26.9 ± 2.8% by the first 500 m compared to rest (*p* < 0.001) and then continued to a nadir of 35.0 ± 2.1% decrease at the end of the row (*p* < 0.01). During the hyperoxic trial, the reduction in SmO_2_ was attenuated to 16.8 ± 3.0% (*p* < 0.01) by the first 500 m and to a nadir of 26.1 ± 2.4% (*p* < 0.01) compared to rest at the end of the 2,000 m row (Figure 3; Table 1), indicating a ~ 25% improvement of SmO_2_ compared to the normoxic trial (*p* = 0.03).

### Neuromuscular Function

**Figure 4:**
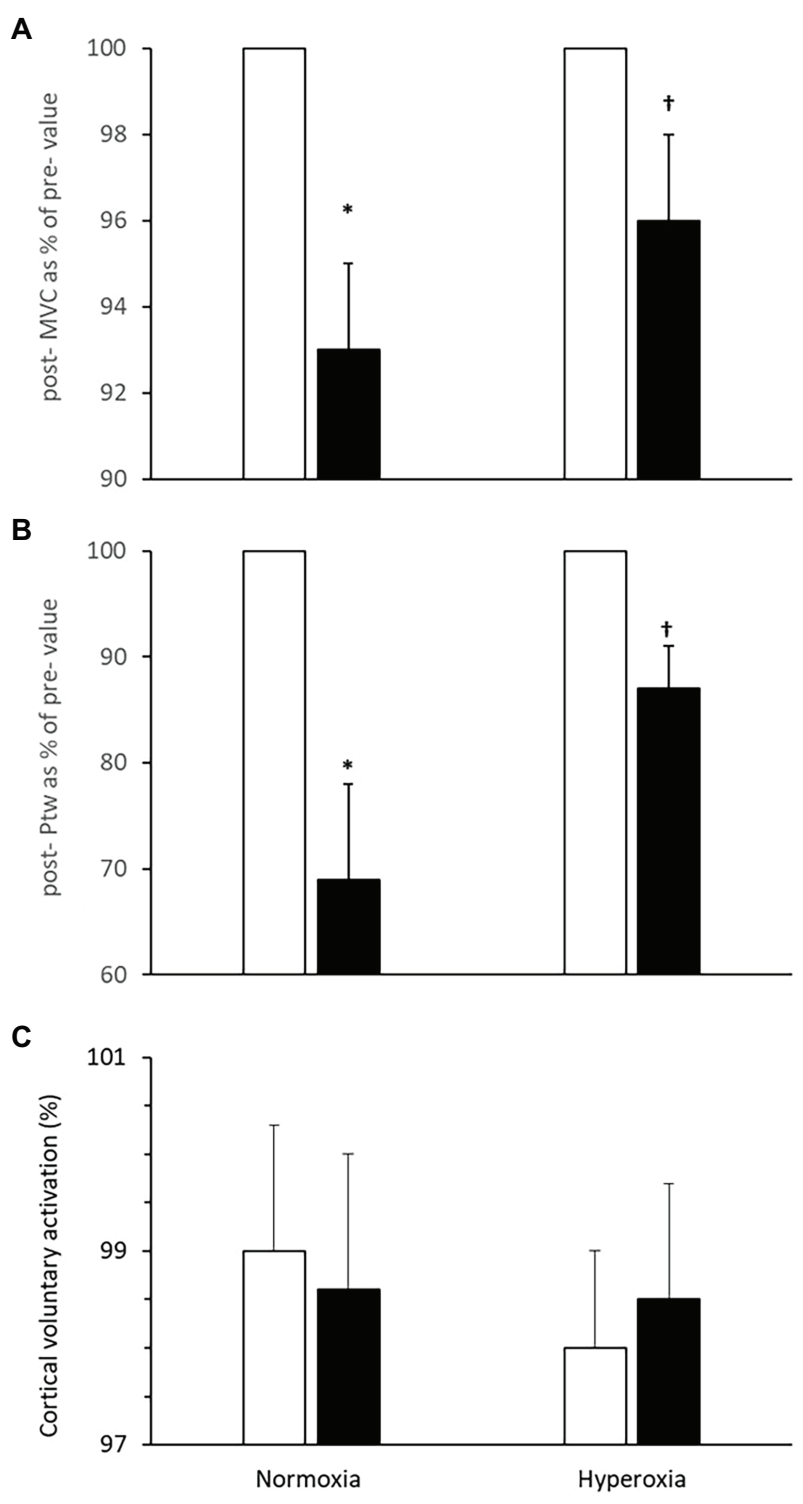
Neuromuscular function pre- (open bars) and immediately post-(closed bars) 2,000 m all-out rowing. **(A)** Maximal voluntary contraction (MVC) of the arm flexor muscles; **(B)** potentiated twitch force (*P*_tw_); and **(C)** cortical voluntary activation. Values are means ± SD, post-values in **(A,B)** are expressed as % of pre-values; *Different from pre-value; †Different from respective value in normoxia (*N* = 11, *p* < 0.05).

There was no significant difference in the MVC prior to the normoxic and hyperoxic trials (502 ± 15 N). There was an interaction [*F*_(1, 6)_ = 6.9, *p* < 0.01] between trials and time for MVC. Following the normoxic trial, there was a reduction in MVC during elbow flexion (7 ± 3%, *p* = 0.014) but following the hyperoxic trial the reduction was not significant (4 ± 3%, *p* = 0.06, Figure 4A).

#### Electrical Stimulation

There was an interaction [*F*_(1, 2)_ = 19.9, *p* < 0.01] between trials and time for (*P*_tw_). The force evoked with direct stimulation of the motor point (*P*_tw_) in biceps brachii at rest immediately after MVC was reduced by 31 ± 9% following the normoxic rowing trial (pre: 140 ± 14 N; post: 95 ± 12 N, *p* = 0.005, Figure 4B). In contrast, after the hyperoxic rowing trial, there was only a ~ 13 ± 4% reduction from the resting value in *P*_tw_ (pre: 148 ± 14 N; post: 128 ± 12 N) that was not statistically significant [*F*_(1, 6)_ = 1.2, *p* = 0.21] and indicative of ~58% attenuation of the deficit observed in the normoxic trial (*p* < 0.05). *P*_tw_ values before the normoxic and hyperoxic trials were similar (*p* = 0.52).

#### Transcranial Magnetic Stimulation

The additional force evoked with superimposed twitch on MVC was not different after rowing in normoxia. Similarly, the size of the superimposed twitch was not different before and after rowing in hyperoxia. VA_TMS_ was not changed after the normoxic trial [pre- 99.0 ± 1.3%; post- 98.6 ± 1.4%; *F*_(1, 6)_ = 1.6, *p* = 0.84] or the hyperoxic trial (pre 98.0 ± 1.0%; post 98.5 ± 1.2%, *p* = 0.88, Figure 4C).

## Discussion

This study evaluated the effect of oxygen supplementation on central and peripheral fatigue of elbow flexor muscles following maximal ergometer rowing in trained male rowers. Maximal rowing in normoxia compromised cerebral and leg muscle oxygenation and reduced arm strength that was associated with reduced force generated with direct electrical stimulation but unaltered force generated with transcranial magnetic stimulation. Supplementation with 30% oxygen in inspired air enhanced arterial oxygen delivery, maintained cerebral oxygenation at resting level and attenuated leg muscle deoxygenation. In addition, hyperoxia attenuated the deficit in arm flexion MVC and force produced with direct electrical stimulation following the normoxic trial, while there was no effect on the force produced with transcranial magnetic stimulation. These findings demonstrate that oxygen supplementation restores cerebral oxygenation and that the attenuation of the arm strength deficit following maximal rowing is associated with peripheral rather than central fatigue mechanisms.

The present finding that arm muscle fatigue following rowing is mainly of peripheral origin is in contrast with the study by Husmann et al. (2017) that evaluated the knee extensors fatigue in elite rowers after intense rowing. Even though Husmann et al. (2017) used peripheral nerve stimulation to assess central fatigue, which provides different information from the cortical stimulation used in the present study (Todd et al., 2004), nevertheless, both methods assess whether the voluntary drive to the muscle is suboptimal for generation of maximal force. Husmann et al. (2017) observed impaired voluntary drive indicating central fatigue with no significant change in indices of peripheral fatigue. A possible explanation for this discrepancy may be offered by the unique synchronous movement pattern of the limbs during rowing. It seems that there is a central constraint that prevents recruitment of the leg muscles during the two-legged exercise inherent to rowing, while the arm muscles are not constrained by such central activation mechanism, and thus, arm muscle fatigue is likely of peripheral origin. Furthermore, such differential fatigue origin for the arms and the legs indicates that leg muscle performance is determined to a large extend by central mechanisms and, combined with the consideration that the largest amount of work during rowing is by far performed by the large leg muscles, it likely explains why an increase in SaO_2_, SmO_2_, and VO_2_max (Nielsen et al., 1998) does not necessarily increase the amount of work performed.

In agreement with the present findings, studies on exercise-induced fatigue that used self-paced whole body exercise of similar duration with the present study (Amann et al., 2006a; Thomas et al., 2015; 5 and 4 km cycling time trials, respectively) suggest that the contribution of central vs. peripheral fatigue is shifted peripherally in short exercise trials (~6 min). In contrast, the observation that the contribution of central vs. peripheral fatigue is shifted centrally with increasing severity of arterial hypoxemia (Amann et al., 2007) suggests central fatigue following maximal rowing (Nielsen et al., 1999). Furthermore, the association of central fatigue with inhibition of slow muscle fiber recruitment (Rasmussen et al., 2007) that are abundant in rowers (Volianitis et al., 2020) supports a contribution of central fatigue to rowing performance. A possible explanation of our finding of a primarily peripheral contribution to arm muscle fatigue may be that exercise-induced fatigue is primarily central below 70–75% SaO_2_ (Amann et al., 2007), while in our study SaO_2_ was attenuated only to 92.5% during rowing in normoxia.

Oxygen supplementation prevented the moderate arterial desaturation (~7%) and maintained SaO_2_ at resting levels. Consequently, peripheral arm fatigue was reduced by more than half, in agreement with the effect of hyperoxia on quadriceps fatigue (Amann et al., 2006b; Romer et al., 2006). However, besides the effect on peripheral muscle fatigue oxygen supplementation increased VO_2_max by ~11% in agreement with previous rowing studies (Peltonen et al., 1995; Nielsen et al., 1999; Volianitis et al., 2008), which is suggestive of central (i.e., cardiovascular) rather than peripheral (i.e., muscular) limitation to rowing performance, albeit work output performed was not increased significantly. Presumably, the restoration of ScO_2_ would attenuate the motor activation limitations associated with cerebral deoxygenation (Rasmussen et al., 2007; i.e., one possible factor of central fatigue) providing support to the notion of central origin to rowing fatigability. On the other hand, the level of cerebral deoxygenation in the present study is small compared with other studies (González-Alonso et al., 2004) suggesting that the subjects could tolerate even higher degree of cerebral deoxygenation. This consideration is supported by that the TMS evaluation did not confirm our presumption of central fatigue in normoxia, and therefore, there was no significant deficit in central motor activation that could be attenuated with oxygen supplementation. It should also be acknowledged that since women exhibit different fatigue characteristics than men (f.x. less fatigable when sustaining a contraction at the same relative intensity; Hicks et al., 2001), our findings on male rowers may not be applicable to the same extent in female rowers.

The O_2_ supplementation also increased MCA *V*_mean_ by ~13% compared to normoxia, a response that can be explained by the higher PaCO_2_ (~2 mmHg) during the hyperoxic trial. However, this elevated MCA *V*_mean_ did not enhance rowing performance by increasing cerebral oxygen delivery, as has been shown also for incremental cycling performance (Smith et al., 2012). Taken together, these findings support the postulate that increasing cerebral oxygen delivery above what is required to maintain cerebral metabolism has little to no positive effect on performance, as expressed by Smith (2016).

The elevation of SmO_2_ with O_2_ supplementation is concomitant with the attenuation of the decline of electrically twitch-evoked force after MVC, suggesting that muscle oxygenation is associated with the attenuation of peripheral fatigue in the hyperoxic trial. Abolishment of the ScO_2_ deoxygenation, while only partial recovery of SmO_2_ deoxygenation, with O_2_ supplementation is in agreement with suggestions that hyperoxia has a larger effect on attenuating the decrease in cerebral rather than muscle oxygenation (Nielsen et al., 1999; Oussaidene et al., 2013). The MVC was reduced following rowing with normoxia but not significantly so with hyperoxia. However, since a MVC is the sum of the motor systems performance including the efficacy of the central nervous system and of the contractile apparatus (Gandevia, 2001), MVC does not allow for separation between central and peripheral (muscle) fatigue.

### Strengths and Limitations

The strength of the present study is the use of an externally valid whole body dynamic exercise model to evaluate neuromuscular fatigue of a muscle group, combined with evaluation of cerebral and muscle oxygenation values providing an integrative depiction of rowing performance and fatigability. The participants are all competitive rowers at national/international level ensuring that the contribution of the muscle group evaluated (arm) to the whole body skilled performance is valid and thus enhance generalization of the findings.

The limitations inherent to evaluations of fatigue post-exercise also apply to our study. The need for a quick measurement post-fatigue is somehow incompatible with the necessity to perform several contractions to provide a reliable evaluation of MVCs or evoked contractions. The elusive nature of fatigue is affected critically by the rapid recovery of most fatigue indices and thus our findings should be interpreted in a relative context applicable to intra-subject comparisons with similar evaluation delay, rather than absolute values and comparisons across subjects or studies. Also, it has to be recognized that the locus of neuromuscular fatigue of a muscle group does not necessarily translate to fatigability in rowing performance.

## Conclusions and Perspectives

In conclusion, the evaluations of ScO_2_, SmO_2_, MCA, and VO_2_ during rowing support a central component to fatigability in agreement with the study by Husmann et al. (2017). Yet, evaluation by electrical and transcranial magnetic stimulation of the elbow flexors indicate peripheral fatigue that is attenuated with O_2_ supplementation. Overall, even though evaluation of some fatigability indices point to a central limitation of rowing performance, that seems not to be the case for the arm muscles.

## Data Availability Statement

The raw data supporting the conclusions of this article will be made available by the authors, without undue reservation.

## Ethics Statement

The studies involving human participants were reviewed and approved by Ethics Committee of Copenhagen (KF 01287471). The patients/participants provided their written informed consent to participate in this study.

## Author Contributions

SV, PR, NP, and NS contributed to the study design, data collection and analysis, and manuscript writing. All authors have read and approved the final version of the manuscript and agreed with the order of presentation of the authors.

## Conflict of Interest

The authors declare that the research was conducted in the absence of any commercial or financial relationships that could be construed as a potential conflict of interest.

## Publisher’s Note

All claims expressed in this article are solely those of the authors and do not necessarily represent those of their affiliated organizations, or those of the publisher, the editors and the reviewers. Any product that may be evaluated in this article, or claim that may be made by its manufacturer, is not guaranteed or endorsed by the publisher.
